# Cardiomyocyte-specific conditional knockout of the histone chaperone HIRA in mice results in hypertrophy, sarcolemmal damage and focal replacement fibrosis

**DOI:** 10.1242/dmm.022889

**Published:** 2016-03-01

**Authors:** Nicolas Valenzuela, Qiying Fan, Faisal Fa'ak, Benjamin Soibam, Harika Nagandla, Yu Liu, Robert J. Schwartz, Bradley K. McConnell, M. David Stewart

**Affiliations:** 1 Department of Biology and Biochemistry, University of Houston, Houston, TX 77204, USA; 2 Department of Pharmacological and Pharmaceutical Sciences, College of Pharmacy, University of Houston, Houston, TX 77204, USA; 3 Department of Computer Science and Engineering Technology, University of Houston-Downtown, Houston, TX 77002, USA; 4 Stem Cell Engineering Department, Texas Heart Institute at St Luke's Episcopal Hospital, Houston, TX 77030, USA

**Keywords:** HIRA, Heart, H3.3, Cardiomyocyte, Histone

## Abstract

HIRA is the histone chaperone responsible for replication-independent incorporation of histone variant H3.3 within gene bodies and regulatory regions of actively transcribed genes, and within the bivalent promoter regions of developmentally regulated genes. The *HIRA* gene lies within the 22q11.2 deletion syndrome critical region; individuals with this syndrome have multiple congenital heart defects. Because terminally differentiated cardiomyocytes have exited the cell cycle, histone variants should be utilized for the bulk of chromatin remodeling. Thus, HIRA is likely to play an important role in epigenetically defining the cardiac gene expression program. In this study, we determined the consequence of HIRA deficiency in cardiomyocytes *in vivo* by studying the phenotype of cardiomyocyte-specific *Hira* conditional-knockout mice. Loss of HIRA did not perturb heart development, but instead resulted in cardiomyocyte hypertrophy and susceptibility to sarcolemmal damage. Cardiomyocyte degeneration gave way to focal replacement fibrosis and impaired cardiac function. Gene expression was widely altered in *Hira* conditional-knockout hearts. Significantly affected pathways included responses to cellular stress, DNA repair and transcription. Consistent with heart failure, fetal cardiac genes were re-expressed in the *Hira* conditional knockout. Our results suggest that transcriptional regulation by HIRA is crucial for cardiomyocyte homeostasis.

## Abstract (precis)

**Summary:** Deletion of *HIRA*, a gene encoding a histone chaperone that lies within the critical region of 22q11.2 deletion syndrome, alters cardiac gene expression and leads to cardiomyopathy.

## INTRODUCTION

Chromatin assembly, the process by which DNA is assembled into nucleosomes, can be grouped into two classifications. Replication-coupled chromatin assembly occurs during S phase as nucleosomes are removed ahead of the replication fork and reassembled on the newly synthesized DNA. Replication-independent chromatin assembly occurs outside of S phase at sites such as remodeled regulatory regions, gene bodies post-transcription, centromeres and telomeres. Octamers of the canonical core histones (H3, H4, H2A and H2B) are utilized for replication-coupled chromatin assembly, whereas histone variants are utilized for replication-independent chromatin assembly. Synthesis of the canonical core histones occurs in a tight window during S phase, whereas histone variants are produced throughout the cell cycle. For recent reviews, see Burgess and Zhang (2013), Gurard-Levin et al. (2014) and Venkatesh and Workman (2015). For this reason, in terminally differentiated cells, such as cardiomyocytes, which have largely exited the cell cycle, histone variants should be utilized for the bulk of chromatin remodeling (Rai and Adams, 2013).

The histone variant H3.3 is the predominant free H3 isoform available for replication-independent chromatin assembly (Ahmad and Henikoff, 2002a). It differs from canonical histone H3 by only four amino acids, which allows it to be utilized by distinct chaperones for chromatin assembly at unique genomic locations. It is enriched at actively transcribed genic regions, regulatory regions and telomeres to facilitate the development of unique chromatin microenvironments with distinct histone modifications (Ahmad and Henikoff, 2002b; Hamiche and Shuaib, 2013; Schwartz and Ahmad, 2005). HIRA is the histone chaperone responsible for replication-independent incorporation of H3.3 at genic regions. In contrast, H3.3 incorporation at most regulatory regions and telomeres is HIRA-independent (Goldberg et al., 2010; Tagami et al., 2004). Thus, HIRA mutants would specifically affect replication-independent assembly of H3.3 across gene bodies, but not affect H3.3 function at telomeres. Although HIRA does not localize to senescence-associated heterochromatin foci (SAHF) – unique regions of facultative heterochromatin that sequester cell cycle genes in senescent cells (Zhang et al., 2007, 2005) – it is necessary for their assembly. HIRA also facilitates chromatin assembly after double-strand-break repair (Adam et al., 2013).

Clinically, the *HIRA* gene is of interest because it lies within the critical region associated with 22q11.2 deletion syndrome (DiGeorge and velocardiofacial syndromes; OMIM 188400) (Halford et al., 1993). Individuals with the 22q11.2 heterozygous deletion exhibit a wide array of developmental, physiological and behavioral abnormalities (Cohen et al., 1999; Ryan et al., 1997; Schneider et al., 2014). Heart defects include an interrupted aortic arch, conotruncal malformation and tetralogy of Fallot (ventricular-septal defect, pulmonary stenosis, right ventricular hypertrophy and overriding aortic arch) (Carotti et al., 2008; Kobayashi et al., 2013; Momma, 2010).

HIRA epigenetically marks active loci through deposition of H3.3, which is the predominant H3 isoform to be modified by the ‘active’ marks of methyl-K4 and acetylation (Ahmad and Henikoff, 2002b; Goldberg et al., 2010; Tagami et al., 2004). For this reason, HIRA should play a major role in defining the gene expression program of any terminally differentiated cell type. Here, we determined the physiological consequence of HIRA deficiency in a single cell type – the cardiomyocyte. Mammalian cardiomyocytes undergo mitotic arrest shortly after birth (Brodsky et al., 1980; Soonpaa et al., 1996; Walsh et al., 2010). For this reason, the adult mammalian heart has extremely limited capacity to regenerate cardiac muscle lost after injury such as myocardial infarction (Beltrami et al., 2001; Malliaras et al., 2013; Senyo et al., 2014). Thus, all chromatin remodeling in cardiomyocytes is replication-independent and HIRA should play a major role in epigenetically defining the cardiomyocyte gene expression program.

The objective of this study was to determine the consequence of ablating replication-independent incorporation of H3.3 on cardiomyocyte gene expression and heart function *in vivo*. Conditional knockout (CKO) of *Hira* in cardiomyocytes of mice widely altered gene expression, including significant downregulation of genes involved in chromatin biology, transcription and DNA repair; fetal cardiac genes were upregulated. Surprisingly, loss of *Hira* did not perturb heart development, but instead resulted in hypertrophy, subepicardial focal replacement fibrosis and altered cardiovascular function. Our results indicate that transcriptional regulation by HIRA is crucial for cardiomyocyte homeostasis.

## RESULTS

### Confirmation of cardiomyocyte-specific *Hira* CKO using *αMyHC-Cre*

Our genetic cross included the *Rosa26^YFP^* allele. Thus, cells subjected to Cre-induced recombination could be monitored by YFP fluorescence. YFP was absent from wild-type (*αMyHC-Cre*-negative) hearts, but distributed throughout the atria and ventricles of control and *Hira* CKO hearts, both of which were positive for the *αMyHC-Cre* transgene (Fig. S1A-C). The *Hira^flox^* allele contains *loxP* sites flanking exon 4. Elimination of exon 4 from *Hira* mRNA transcripts was tested by reverse transcription PCR using primers flanking exon 4. The wild-type transcript was detected in wild-type and *Hira* CKO hearts. Deleted exon 4 transcripts were only detected in *Hira* CKO hearts (Fig. S1D). These results confirm correct expression of Cre and functionality of the *Hira* CKO allele.

### Loss of HIRA results in focal replacement fibrosis

**Fig. 1.**
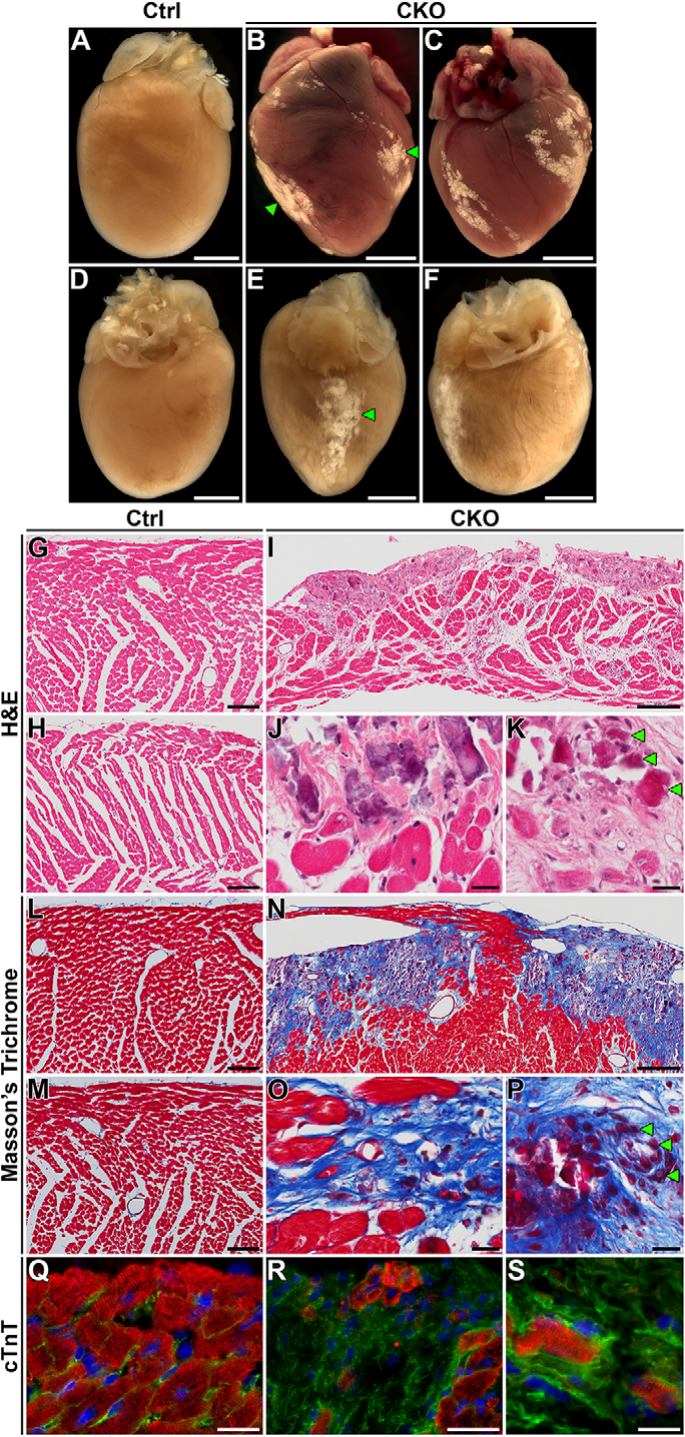
**Loss of HIRA results in focal replacement fibrosis.** (A-F) Stereoimages of whole hearts from 6-month-old mice post-perfusion (A,D-F) or 6-week-old mice without perfusion (B,C). Hearts of control mice (*αMyHC-cre^Tg/+^; Hira^flox/+^*) appeared normal (A,D). Hearts of *Hira* CKO mice (*αMyHC-cre^Tg/+^; Hira^flox/−^*) exhibited visible white scarring on the surface at 6 weeks (B,C) and 6 months (E,F) of age (green arrowheads). (G-K) Ventricular sections from 6-month-old mice stained with H&E. Loss of cardiomyocytes within the surface lesions of *Hira* CKOs were obvious. (L-P) Masson's trichrome-stained ventricular sections from 6-month-old mice. *Hira* CKO hearts exhibited collagen deposition (fibrosis) within the lesion (blue stain). Degenerating cardiomyocytes were apparent throughout the scar (green arrowheads in K and P). (Q-S) Immunofluorescence against cardiac troponin T (cTnT) in 6-week-old hearts, illustrating the presence of cardiomyocytes within the fibrotic lesions [red, cTnT; green, membranes (WGA-488); blue, nuclei (DAPI)]. Scale bars: 2 mm (A-F), 200 µm (I,N), 100 µm (G,H,L,M,Q,R) and 20 µm (J,K,O,P,S). Ctrl, control; CKO, *Hira* conditional knockout.

Based on the crucial role for HIRA in epigenetically marking active loci by deposition of variant histone H3.3, we hypothesized that loss of HIRA would be detrimental to cardiomyocyte gene expression and produce congenital heart defects. To our surprise, heart development proceeded normally in *Hira* CKO mice. We found no evidence for embryonic or neonatal lethality (Table S1). However, examination of *Hira* CKO hearts at 6 weeks and 6 months of age revealed striking white surface scars (Fig. 1A-F). These lesions preferentially localized to the subepicardial region of the ventricular free walls. Histological examination of these macroscopic lesions showed that they contained degenerating cardiomyocytes and a large degree of collagen deposition (Fig. 1G-P). Sparse cardiac troponin-T-positive cardiomyocytes could still be detected within the fibrotic areas (Fig. 1Q-S). Collectively, elimination of HIRA produced a pathology that is consistent with focal replacement fibrosis preferentially localized to the subepicardial myocardium.

### Compromised sarcolemmal integrity

**Fig. 2.**
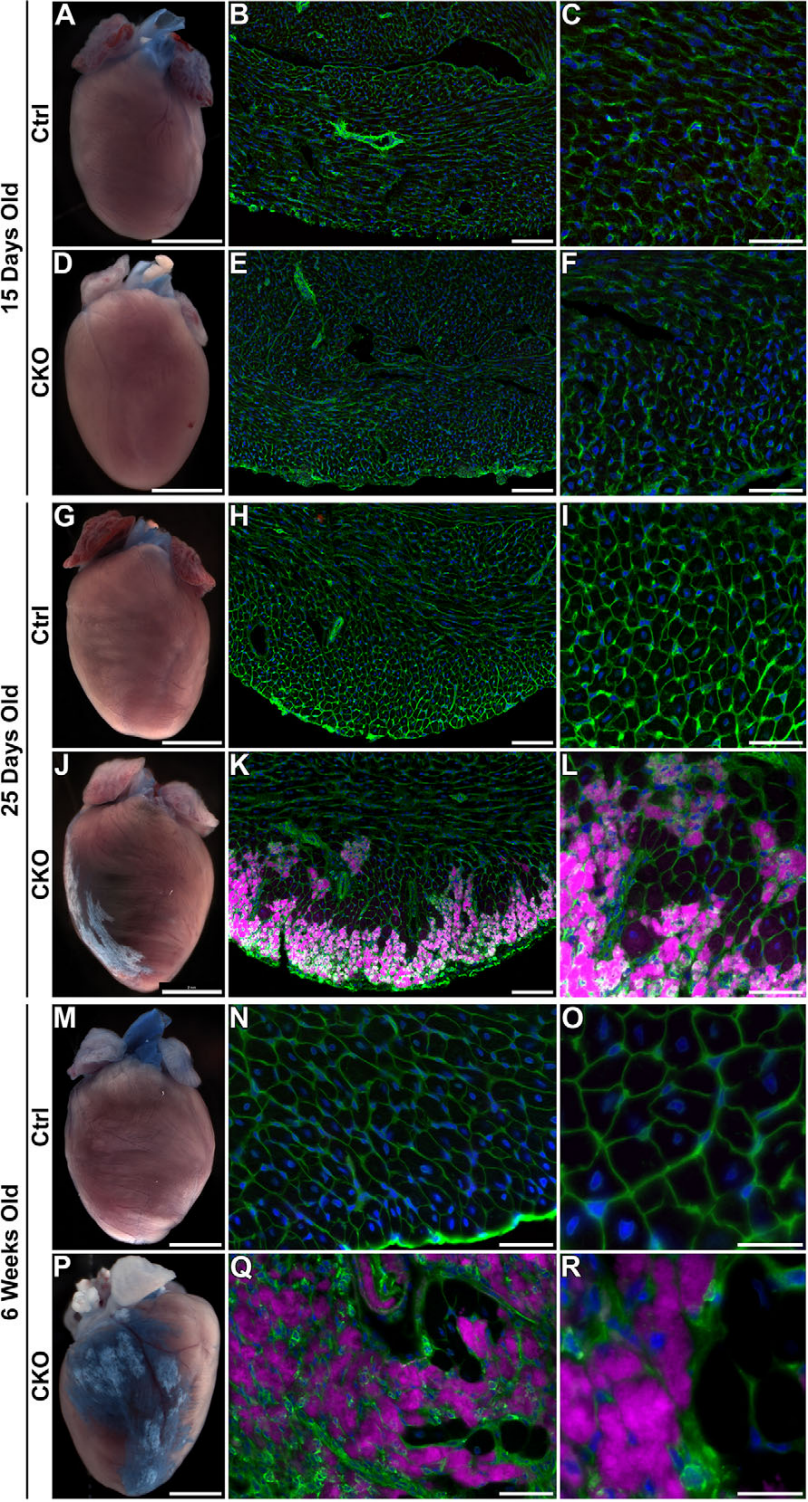
**Compromised sarcolemmal integrity.** Sarcolemmal damage was assayed *in vivo* by uptake of Evans blue dye (EBD). EBD-positive cardiomyocytes were visualized by brightfield stereoimaging and by far-red fluorescence in ventricular cryosections. (A-F) At 15 days of age, no EBD was detectable in the ventricles of control or *Hira* CKO hearts. (G-L) By 25 days of age, visible surface scars were obvious and EBD-positive cardiomyocytes were detectable in *Hira* CKO hearts, but not controls. (M-R) 6-week-old *Hira* CKO mice exhibited EBD-positive cardiomyocytes surrounding the fibrotic lesions. Green, membranes (WGA-488); purple, EBD; blue, nuclei (DAPI). Scale bars: 2 mm (A,D,G,J,M,P), 100 µm (B,E,H,K), 50 µm (C,F,I,L,N,Q) and 20 µm (O,R). Ctrl, control; CKO, *Hira* conditional knockout.

The focal replacement fibrosis seemed to be caused by local degeneration of cardiomyocytes. Thus, we assayed for compromised sarcolemmal integrity as an early sign of potential degeneration by uptake of Evans blue dye (EBD). EBD was administered 18 h prior to sacrifice. At 15 days of age, we found no EBD-positive cardiomyocytes in either control or *Hira* CKO hearts (*n*≥6 mice/group) (Fig. 2A-F). By 25 days of age, EBD-positive cardiomyocytes were detectable in four out of five *Hira* CKOs, three of which exhibited visible surface scars on the right ventricle free wall. No EBD-positive cardiomyocytes were detectable in control animals (*n*=10) (Fig. 2G-L). By 6 weeks of age, all (*n*=3) *Hira* CKOs exhibited regions of EBD-positive myocardium, which surrounded the surface scars. Again, no EBD-positive cardiomyocytes were detected in control animals (Fig. 2M-R). These data indicate that cardiomyocyte sarcolemmal integrity becomes compromised in focal subepicardial areas between 15 and 25 days after birth. Sarcolemmal perforation seems to precede cardiomyocyte degeneration and fibrotic scar formation.

### Impaired cardiac function and hypertrophy

**Fig. 3.**
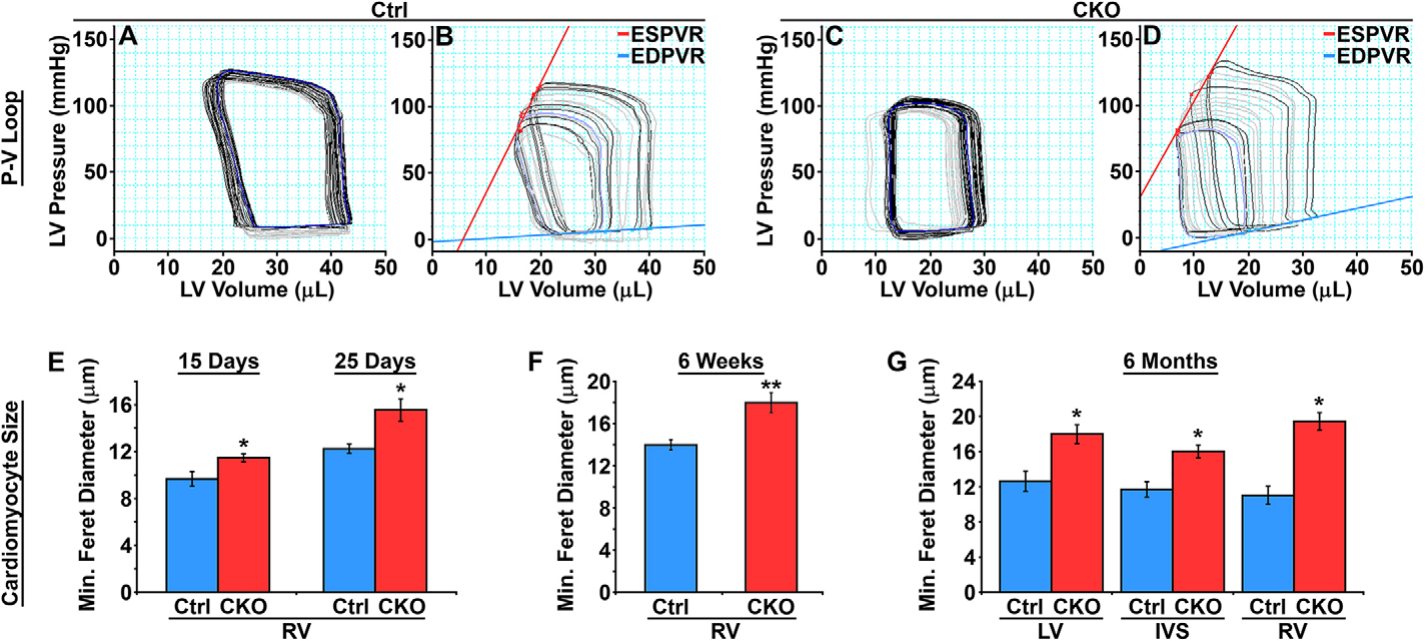
**Impaired cardiac function.** (A-D) *In vivo* assessment of contractility with left-ventricular pressure-volume (P-V) relationships. P-V loops were recorded under baseline conditions (A,C) and during inferior vena cava (IVC) occlusions to decrease the preload (B,D). Representative P-V loops under baseline for control (A) and *Hira* CKO (C), and under IVC occlusions for control (B) and *Hira* CKO (D) mice (*n*=7 mice/group). (E-G) Hypertrophic cardiomyocytes in *Hira* CKO hearts. Cardiomyocyte size was quantified by minimum Feret diameter measurements. (E,G) ANOVA, *P<0.001, *n*≥6 (15 days old) or 5 (25 days and 6 months old). (F) Student's *t*-test, **P<0.01, *n*≥5 animals/group. Ctrl, control; CKO, *Hira* conditional knockout; EDPVR, end-diastolic pressure-volume relationship; ESPVR, end-systolic pressure-volume relationship; IVS, interventricular septum; LV, left ventricle; RV, right ventricle.

In order to understand whether HIRA affects cardiac function *in vivo*, we performed pressure-volume (P-V) loop measurements on both control and *Hira* CKO mice at 6 months of age. Representative P-V loop results are illustrated in Fig. 3A-D and hemodynamic parameters are reported in Table 1. Compared to control mice, *Hira* CKO mice showed significantly increased arterial elastance (Ea), which is a measure of arterial load. Decreased stroke volume (SV), stroke work (SW) and cardiac output (CO) were also observed in *Hira* CKO mice. The slope of the end-diastolic pressure-volume relationship (EDPVR), which describes diastolic function and the passive properties of the ventricle, was elevated in *Hira* CKO mice with no significant effect to the slope of end-systolic pressure-volume relationship (ESPVR; E_es_/E_max_, end-systolic elastance), which describes the maximal developed ventricular pressure at any given ventricular volume. This indicates an increase in diastolic myocardial stiffness in *Hira* CKO mice. *Hira* CKO mice also exhibited increased dp/dt_max_-EDV (relationship between peak rate of pressure rise and EDV), which describes ventricular contractile performance. Each of these P-V loop hemodynamic parameters, EDPVR, ESPVR and dp/dt_max_–EDV, offer the unique advantage of providing insights into cardiac function that are independent of inherent variables of preload and heart rate, thus providing precise measurements of ventricular performance. Collectively, these data indicate impaired cardiac function in the absence of HIRA.

**Table 1.**
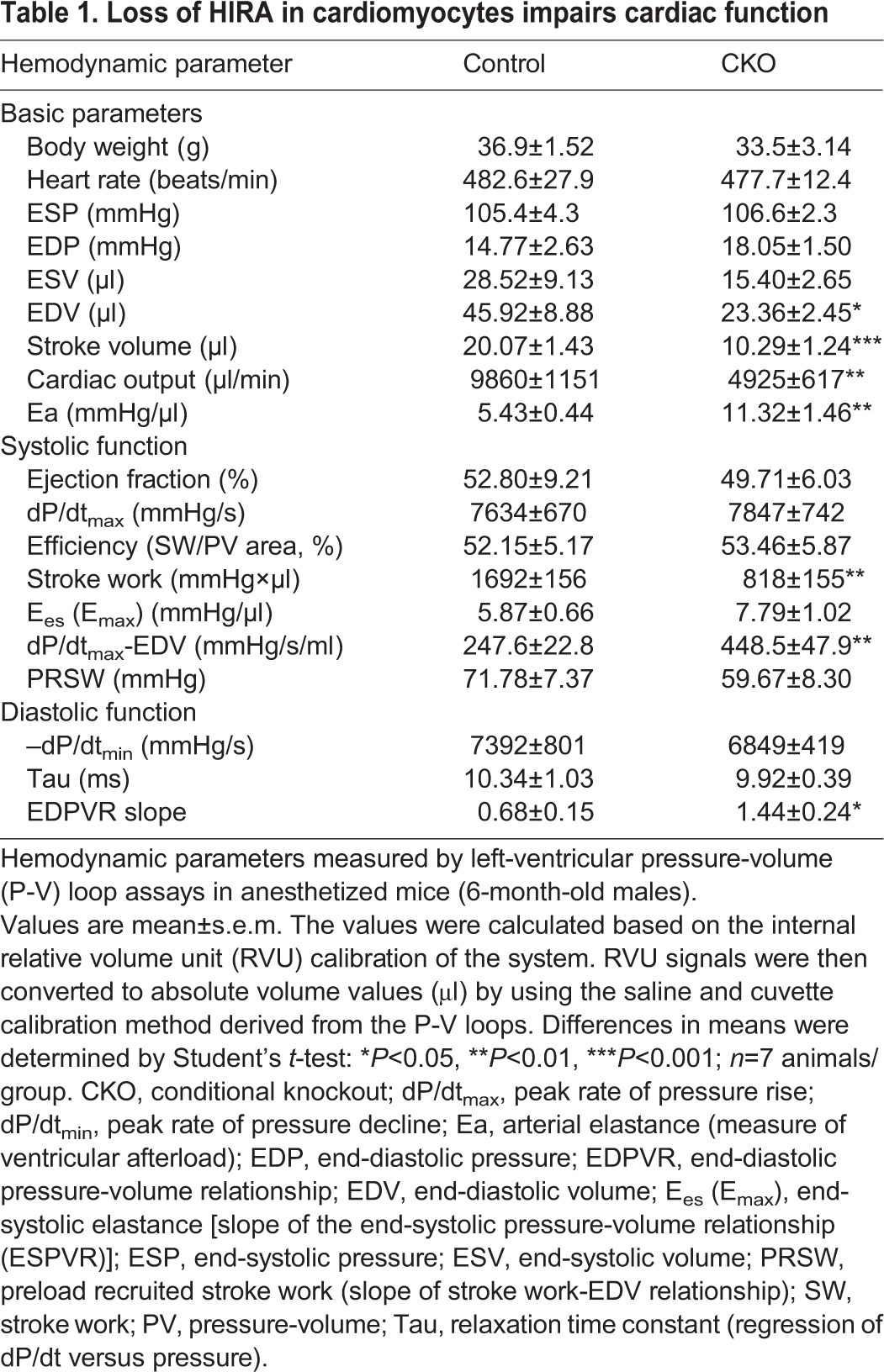
**Loss of HIRA in cardiomyocytes impairs cardiac function**

*Hira* CKO mice also displayed cardiomyocyte hypertrophy as assayed by measuring cardiomyocyte minimum Feret diameter. Hypertrophic cardiomyocytes were detectable in the right ventricle free wall as early as 15 days after birth (Fig. 3E), which is prior to the appearance of sarcolemmal damage. Measurements at 25 days (Fig. 3E), 6 weeks (Fig. 3F) and 6 months (Fig. 3G) of age also revealed hypertrophy. These data indicate that hypertrophy is an early consequence of HIRA deficiency and implicate HIRA in the regulation of the hypertrophic response.

### Loss of HIRA did not cause aberrant proliferation of cardiomyocytes or apoptosis

Several reports have implicated HIRA in cell cycle arrest, senescence and tumor suppression (Hall et al., 2001; Rai et al., 2014; Zhang et al., 2005). For this reason, we tested the idea that loss of HIRA led to aberrant activation of the cell cycle in cardiomyocytes. To this end, we treated 6-week-old mice with BrdU 4 h prior to sacrifice and assayed cell proliferation by anti-BrdU immunofluorescence. The results of this assay revealed zero BrdU-positive cardiomyocytes in both control and *Hira* CKO hearts. The only BrdU-positive cells were interstitial cells and cells within the fibrotic lesions (Fig. S2A-C). Neonatal (postnatal day 2) ventricular myocardium was used as a positive control for proliferating cardiomyocytes (Fig. S2D).

Next, we tested the idea that cardiomyocyte degeneration was the consequence of apoptosis. Apoptosis was assayed by TUNEL in 6-week-old mice. These data revealed zero TUNEL-positive cardiomyocytes in either control or *Hira* CKO hearts. The only TUNEL-positive cells were interstitial cells and a few cells within the fibrotic scars (Fig. S2E-G). Thus, loss of HIRA did not promote cardiomyocyte apoptosis.

### Effect of HIRA deficiency on the cardiac transcriptome

HIRA is best known for its role in epigenetically marking transcribed genes through replication-independent assembly of nucleosomes containing variant histone H3.3. Thus, we hypothesized that HIRA plays an important role in defining the cardiac transcriptome. To determine the consequence of HIRA deficiency on cardiac gene expression, we compared gene expression in control and *Hira* CKO hearts at 6 weeks of age by microarray using mRNA extracted from non-scarred free-wall left-ventricular tissues. Appropriate grouping of control and *Hira* CKO data was confirmed by principal component analysis (PCA) and clustering analyses (Fig. S3). A heat map of all differentially expressed genes is presented in Fig. S3B. Loss of HIRA resulted in the upregulation of 269 genes and downregulation of 128 genes. Gene ontology analysis of downregulated transcripts revealed the most significantly affected biological processes to be those related to responses to cellular stress, chromatin metabolism and transcriptional regulation. Fewer gene ontology terms were associated with upregulated genes, with little in common between the terms (Table 2). In broad terms, the results of our gene expression profiling suggested that cardiomyocyte degeneration and cardiomyopathy were either due to impaired stress responses or misregulation of transcription.

**Table 2.**
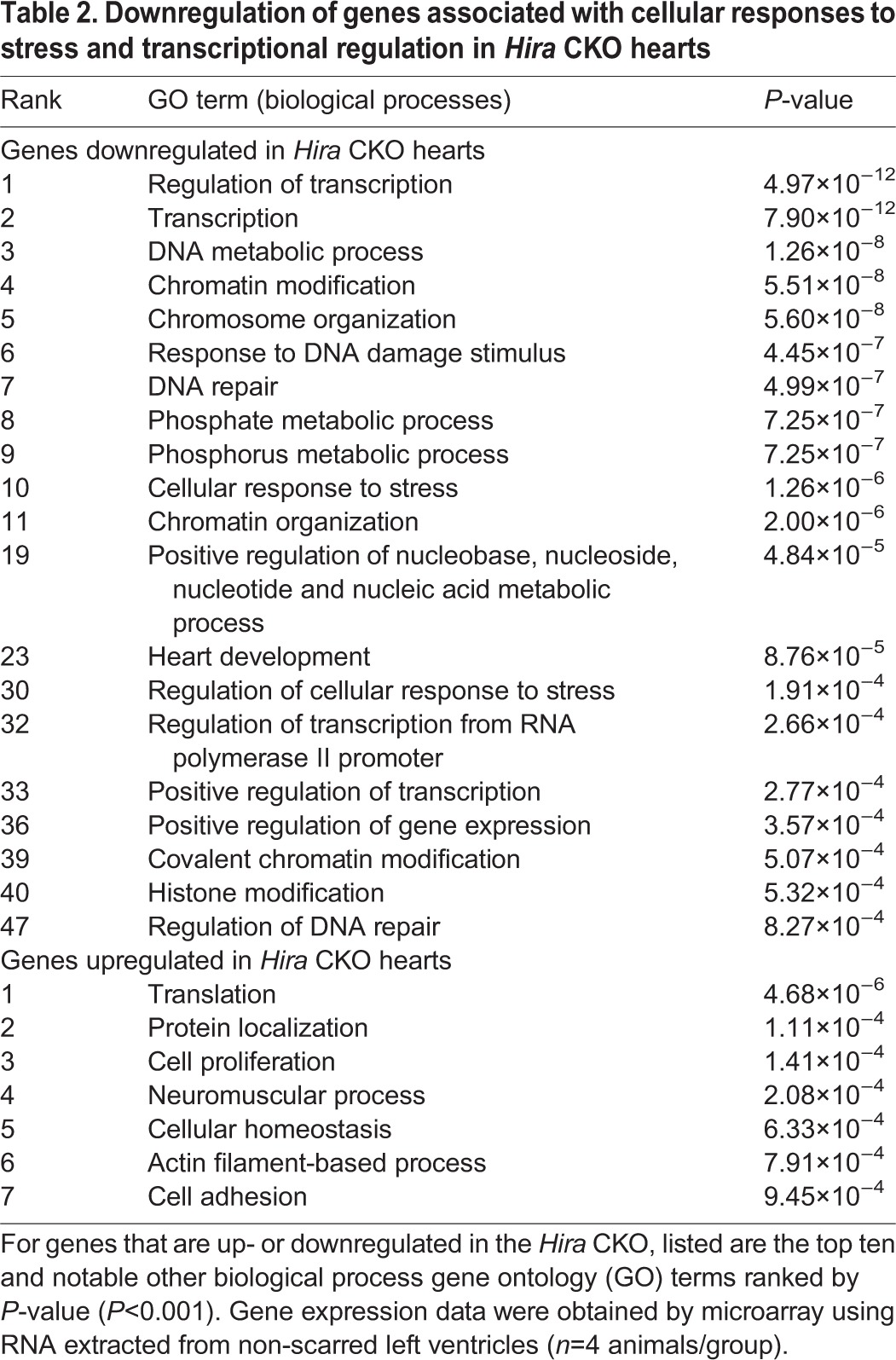
**Downregulation of genes associated with cellular responses to stress and transcriptional regulation in *Hira* CKO hearts**

We specifically interrogated the microarray results for genes associated with striated muscle (Fig. S4). Several developmental transcription factors and contractile proteins were upregulated, including *Nkx2-5*, *Tbx2* and *Myh7/β-MyHC*. Notable downregulated genes included cardiac transcriptional regulators (*Zfpm2*, *Hand2*, *Taz*, *Csrp3*, *Mef2d* and *Chd7*), myofibril assembly factors (*Ttn* and *Tcap*) and adrenergic α1 receptors (*Adra1a* and *Adra1b*).

The *Hira* CKO phenotype is similar to that reported for desmin (*Des*) knockout mice, which exhibit subepicardial fibrosis that predominantly localizes to the free wall of the right ventricle (Thornell et al., 1997). For this reason we specifically looked for alterations in expression of desmosome- or intermediate-filament-associated genes (Fig. S5). Expression of *Des* was unaffected. Upregulated genes linked to cardiomyopathy included *Pkp2*, *Dst*, *Dsg2* and *Jup*; downregulated genes included *Pkp1*, *Dsg1a*, *Pkp4*, *Vcl*, *Gja1* and *Ctnna1*.

### Reduced expression of DNA repair genes, but no evidence for increased DNA damage

The results of our gene expression profiling showed broad misregulation of DNA repair genes, with 90 genes downregulated and 45 upregulated in the *Hira* CKO (Fig. S6). Thus, we tested whether *Hira* CKO mice exhibited increased DNA damage by immunolocalization of γ-H2A.X. A low level of γ-H2A.X immunoreactivity was detectable in all cardiomyocytes of both control and *Hira* CKO mice (Fig. S2H,I). *Hira* CKO hearts displayed some γ-H2A.X-positive nuclei within the scar regions (Fig. S2J). Based on morphology, these γ-H2A.X-positive cells were not cardiomyocytes. As mentioned above, we did not detect any TUNEL-positive cardiomyocyte nuclei in the *Hira* CKO either (Fig. S2E-G). In summary, although loss of HIRA caused misregulation of many genes associated with DNA repair, we found no evidence for increased DNA damage in the *Hira* CKO.

### Minor evidence for increased oxidative stress

**Fig. 4.**
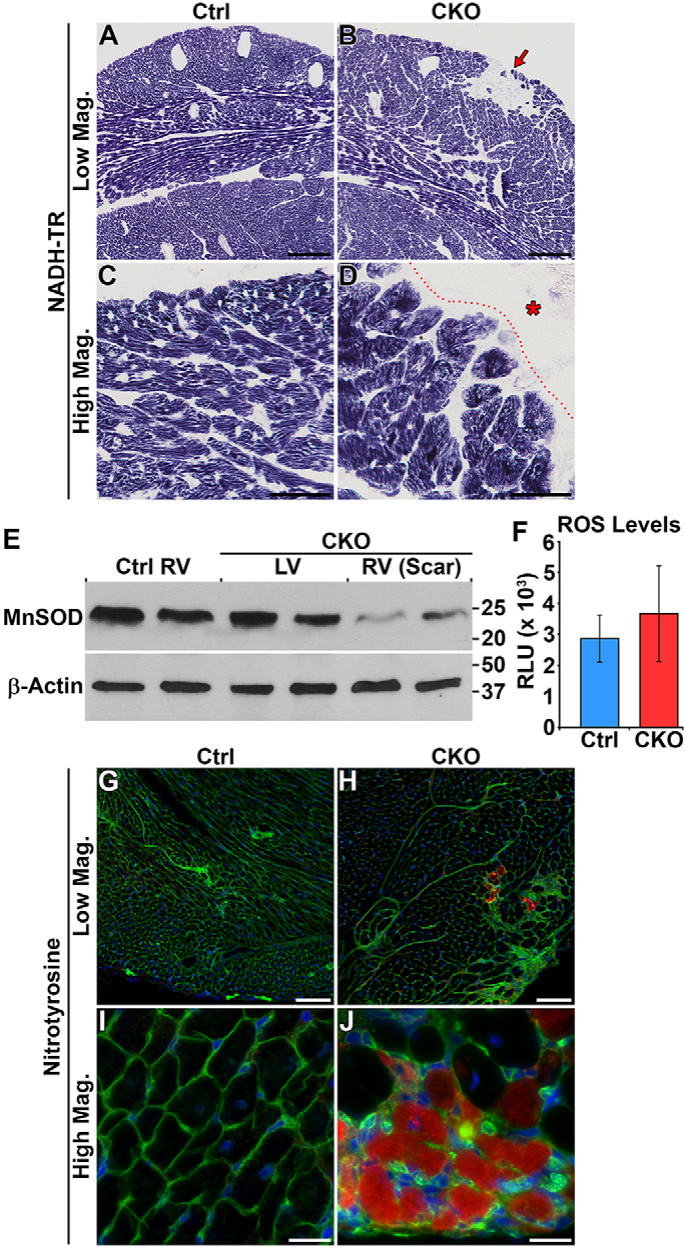
**Minor evidence for increased oxidative stress.** (A-D) NADH-TR staining. Mitochondrial content was equivalent throughout the free wall of the right ventricle and septum for both control and *Hira* CKO mice (A,B). Oxidative staining was notably absent from scar regions (red arrow in B, red asterisk in D). *n*=6 mice/group. The dotted line in D demarcates the border of the scar. (E) Western blot for manganese superoxide dismutase (MnSOD), an indicator of cellular response to oxidative stress. Each lane contains protein extract from an individual animal. MnSOD protein levels were equivalent between control right ventricle and *Hira* CKO left ventricle, but reduced in *Hira* CKO right ventricle, which harbored the scar regions. (F) There was no difference in ventricular ROS accumulation as determined by conversion of 2′,7′-dichlorodihydrofluorescein to 2′7′-dichlorofluorescein. *n*=3 mice/group. (G-J) Immunofluorescence for nitrotyrosine. Nitrotyrosine-positive cardiomyocytes (red) were absent from control hearts (G,I). Two out of six *Hira* CKO hearts exhibited small patches of nitrotyrosine-positive cardiomyocytes (H,J). *n*=6 mice/group. Green, membranes (WGA-488); blue, nuclei (DAPI). Scale bars: 200 µm (A,B), 50 µm (C,D,G,H) and 20 µm (I,J). Ctrl, control; CKO, *Hira* conditional knockout; Mag, magnification; RLU, relative light units; ROS, reactive oxygen species; RV, right ventricle; LV, left ventricle.

Loss of HIRA resulted in the misregulation of many genes associated with the oxidative stress response (Fig. S7). Thus, we tested for signs of increased oxidative stress in *Hira* CKO cardiomyocytes at 6 weeks of age. Mitochondrial content, as assayed by nicotinamide dehydrogenase tetrazolium reductase (NADH-TR) oxidative staining, was equivalent throughout the ventricular walls and septum of control and *Hira* CKO mice (Fig. 4A-D). However, NADH-TR staining was notably absent from scar regions owing to degeneration of cardiomyocytes and focal replacement fibrosis in these regions (Fig. 4B,D). Western blots were performed to test protein levels of manganese superoxide dismutase (MnSOD), which is normally upregulated in response to oxidative stress. To our surprise, MnSOD levels were equivalent between control free-wall right ventricle and non-scarred *Hira* CKO left ventricle, but significantly reduced in scar-containing *Hira* CKO free-wall right ventricle (Fig. 4E). Reduced MnSOD in scar regions is likely due to cardiomyocyte degeneration and focal replacement fibrosis, but could also signal an impaired response to oxidative stress.

Next, we assayed accumulation of reactive oxygen species (ROS) by conversion of nonfluorescent 2′,7′-dichlorodihydrofluorescein to fluorescent 2′7′-dichlorofluorescein using whole-cell extracts from non-scarred ventricular tissues (right and left combined). These data revealed no difference in ROS content between control and *Hira* CKO hearts (Fig. 4F). Next, we indirectly tested for elevated ROS by immunolocalization of 3-nitrotyrosine, a stable end-product of oxidation by reactive nitrogen species. Nitrotyrosine-positive cardiomyocytes were completely absent from control hearts (Fig. 4G,I). A few small patches of nitrotyrosine-positive cardiomyocytes were detected in two out of six *Hira* CKO hearts, usually in proximity to fibrotic areas (Fig. 4H,J). Collectively, these data indicate altered expression of oxidative stress genes in the *Hira* CKO, but very minor increases in ROS content. Thus, cardiomyocyte degeneration in the *Hira* CKO is unlikely to be caused by increased oxidative stress.

### Re-expression of fetal cardiac genes

**Fig. 5.**
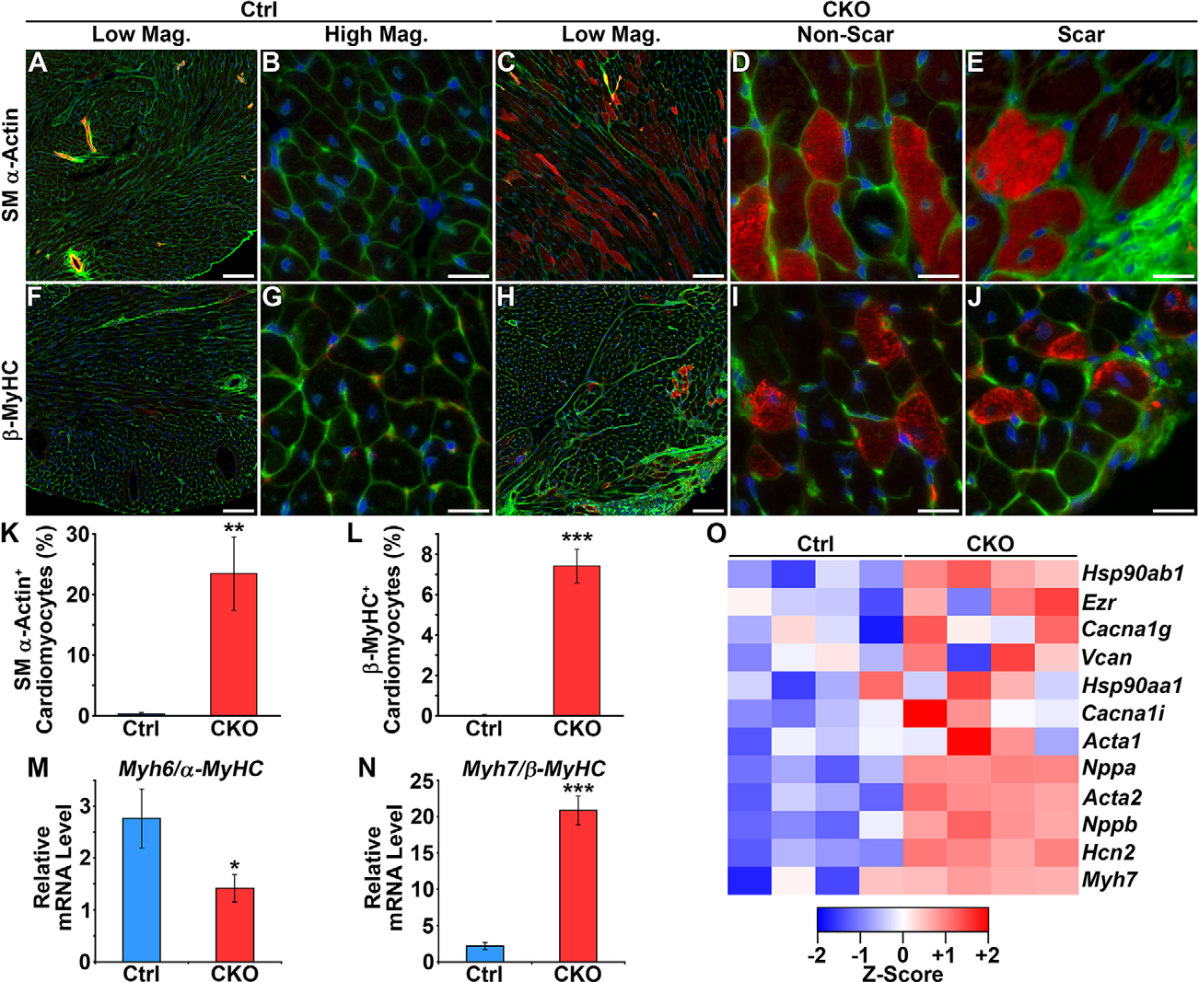
**Re-expression of fetal cardiac genes.** (A-E) Immunofluorescence for smooth-muscle α-actin. Smooth-muscle α-actin (red) was absent from control hearts (A,B), but expressed in *Hira* CKO cardiomyocytes (C-E). (F-J) Immunofluorescence for β-myosin heavy chain (β-MyHC). β-MyHC (red) was absent from control hearts (F,G), but expressed in *Hira* CKO cardiomyocytes (H-J). Green, membranes (WGA-488); blue, nuclei (DAPI). (K) 23.5% of *Hira* CKO cardiomyocytes were positive for smooth-muscle α-actin. (L) 7.4% of *Hira* CKO cardiomyocytes were positive for β-MyHC. (M,N) Real-time PCR gene expression assays. *Myh6* (α-MyHC) expression was reduced and *Myh7* (β-MyHC) expression was increased in *Hira* CKO hearts. For all assays, *n*=6 mice/group. Student's *t*-test; *P<0.05, **P<0.01, ***P<0.001. (O) Upregulation of fetal cardiac genes in *Hira* CKO hearts as determined by microarray (*n*=4 mice/group; *P*<0.05). Color key indicates log2 fold-change. Scale bars: 100 µm (A,C,F,H) and 20 µm (B,D,E,G,I,J). Ctrl, control; CKO, *Hira* conditional knockout; SM, smooth muscle; MyHC, myosin heavy chain; Mag, magnification.

The fetal cardiac gene program is normally silenced shortly after birth, but is re-expressed during heart failure (Dirkx et al., 2013). Thus, we tested whether loss of HIRA promoted fetal gene expression. As expected, control hearts exhibited little to no smooth-muscle α-actin- or β-MyHC-positive cardiomyocytes (Fig. 5A,B,F,G). In contrast, *Hira* CKO hearts exhibited widespread smooth-muscle α-actin-positive cardiomyocytes (Fig. 5C-E) and focal regions of β-MyHC-positive cardiomyocytes (Fig. 5H-J). Quantification of these results is presented in Fig. 5K,L. At the mRNA level, *Hira* CKO hearts exhibited reduced expression of *α-MyHC*(*Myh6*) and increased expression of *β-MyHC *(*Myh7*)** (Fig. 5M,N). Microarray results showed expression of most heart-failure-associated fetal genes to be higher in the *Hira* CKO (Fig. 5O). These results support the idea that HIRA is necessary for correct transcriptional regulation in cardiomyocytes, perturbation of which results in heart failure.

## DISCUSSION

Individuals with 22q11.2 deletion syndrome (DiGeorge and velocardiofacial syndromes) are heterozygous for a genomic deletion that includes the *HIRA* gene. This syndrome affects one in 4000 births, equally affecting males and females (Devriendt et al., 1998). *HIRA* falls within the smaller critical 1.5 Mb region that is deleted in 10% of cases (Carlson et al., 1997). Thus, *HIRA* is a candidate for contributing to the symptoms of the disease. Loss of the *HIRA* gene is widely used for cytogenetic testing by fluorescent *in situ* hybridization (Rauch et al., 2005; Wozniak et al., 2010). Heart defects resulting from 22q11.2 include an interrupted aortic arch, conotruncal heart malformations and tetralogy of Fallot. In chick embryos, knockdown of *Hira* in the neural crest causes persistent truncus arteriosus (Farrell et al., 1999). Although *Hira* heterozygous mice are phenotypically normal with no apparent congenital heart defects, cardiomyopathy and heart failure resulting from total loss of HIRA in cardiomyocytes gives insight into the pathways affected by reduction in HIRA in individuals with 22q11.2. Such individuals might be susceptible to hypertrophy, sarcolemmal damage and focal replacement fibrosis if an additional mutation occurred in the remaining *HIRA* allele or in a gene in a HIRA-interacting pathway.

The earliest abnormality resulting from loss of *HIRA* that we found was cardiomyocyte hypertrophy, detectable by postnatal day 15. Hypertrophy preceded sarcolemmal damage, which was first seen at postnatal day 25. These data indicate that HIRA might inhibit the hypertrophic response. Thus, individuals harboring the 22q11.2 deletion might be susceptible to hypertrophy, not just owing to pulmonary stenosis, but due to intrinsic cardiomyocyte defects. These data prompted us to question whether HIRA expression is reduced in hypertrophic or heart-failure conditions. We searched public gene expression datasets, but found no evidence for alterations in *HIRA* mRNA levels. However, we cannot discount the notion that HIRA protein levels might be reduced in these models. A recent report illustrated that HIRA protein levels can vary independently of mRNA (Majumder et al., 2015).

*Hira* CKO cardiomyocytes are susceptible to compromised sarcolemmal integrity. This was evident in EBD uptake assays. EBD-positive cardiomyocytes surrounded fibrotic areas, but were also present in small patches free from fibrosis. It seems that sarcolemmal damage precedes degeneration and these foci of EBD-positive cardiomyocytes represent regions that will eventually develop focal replacement fibrosis. Sarcolemmal permeability impairs excitation-contraction coupling, which could lead to all of the pathological characteristics of the *Hira* CKO phenotype, including cardiomyocyte hypertrophy, degeneration, fibrosis and heart failure.

Based on the known cellular processes for which HIRA's functions are important, there are several potential mechanisms underlying heart failure in the *Hira* CKO. Because HIRA is responsible for H3.3 deposition at actively transcribed loci, effectively epigenetically marking active genes, loss of HIRA could impair cardiac gene expression by lowering transcription of all genes actively transcribed by RNA polymerase II. However, our microarray data do not support this idea because more genes were upregulated than downregulated. These results argue against general reduced transcription as the sole cause of cardiomyopathy in the absence of HIRA. However, because HIRA is widely utilized for transcriptional regulation and H3.3 enrichment occurs not just at active genes, but also at developmentally regulated silenced genes (Goldberg et al., 2010), HIRA deficiency could both positively and negatively affect gene expression in a context-dependent manner. This idea fits with the results of our microarray, in which a large set of genes was upregulated and a large set of genes was downregulated. It is also possible that genes are not expressed or repressed to the correct degree. Additionally, transcriptional responses to intracellular signaling pathways might be muted in the absence of HIRA. In addition to transcribed regions, HIRA also remodels chromatin with H3.3-containing nucleosomes at some regulatory regions. H3.3 at these regions is thought to provide a dynamic chromatin environment, which increases accessibility to trans-acting factors (Banaszynski et al., 2013). Therefore, loss of HIRA could negatively impact transcriptional regulation by trans-acting factors at specific cardiac genes. Indeed, we found re-expression of the fetal gene program in the *Hira* CKO, increased TGFβ pathway genes and decreased expression of α1 adrenergic receptors, all of which contribute to pathological remodeling.

Loss of HIRA does not completely ablate H3.3 incorporation in all cases. For example, H3.3 incorporation at the *Runx1* enhancer was not completely lost in *Hira*-null embryonic stem (ES) cells (Majumder et al., 2015). Thus, in addition to HIRA, other factors contribute to replication-independent H3.3 assembly. It remains to be determined to what extent H3.3 incorporation was disrupted in *Hira* CKO cardiomyocytes. Intriguingly, HIRA is also necessary for the nuclear localization of RUNX1 (Majumder et al., 2015). This observation opens the possibility that some cardiac transcription factors might not have proper access to their target genes in *Hira* CKO cardiomyocytes.

HIRA is required for transcription recovery after DNA damage repair by depositing H3.3 into newly assembled chromatin (Adam et al., 2013). Thus, cardiomyocyte degeneration in the *Hira* CKO could be the result of failed transcription restart after DNA repair. However, we did not strictly observe downregulation of gene expression, which would indicate impaired transcription restart. We found no evidence for increased DNA damage in *Hira* CKO cardiomyocytes by immunolocalization of γ-H2A.X or TUNEL assays; however, many genes in the DNA repair pathway were misregulated (Fig. S6). Thus, HIRA might be necessary for efficient regulation of DNA repair genes in response to insults such as oxidative stress.

Cardiomyocytes are subject to a high degree of oxidative stress, which is a leading cause of cardiac injury (Victorino et al., 2015). Failure to appropriately respond to oxidative stress presumably would cause cardiomyocyte degeneration as observed in the *Hira* CKO. As would be expected for localized oxidative-stress-induced damage, *Hira* CKO fibrotic lesions were located in isolated subepicardial patches, rather than evenly dispersed throughout the myocardium. Expression of oxidative-stress response genes was widely altered in the *Hira* CKO (Fig. S7), supporting the idea that loss of HIRA impaired these pathways. However, we found little evidence for increased ROS levels in the *Hira* CKO. ROS assays showed no effect of HIRA loss, but small patches of nitrotyrosine-positive cardiomyocytes were observed in some *Hira* CKO hearts in the vicinity of the scar. MnSOD protein levels, an indicator of the oxidative stress response, were decreased in the region of the right ventricle harboring the surface lesion. Decreased MnSOD protein could be the result of cardiomyocyte death, which in turn would result in fewer mitochondria and less MnSOD. However, this explanation is unlikely to account for the great decrease in MnSOD protein detected by western blot. In western blots, MnSOD levels in the area containing the lesion were reduced by at least 80%. In contrast, the volume of the focal fibrosis accounted for no more than 10% of the tissue used for protein extraction. Furthermore, NADH-TR stains revealed equivalent oxidative stain intensity in cardiomyocytes near to or away from the lesions. Thus, cardiomyocytes in the vicinity of the lesion do not have significantly fewer mitochondria. Collectively, these data suggest that the oxidative stress response is impaired in the absence of HIRA even though ROS levels are not significantly elevated.

The focal replacement fibrosis observed in the *Hira* CKO is similar to that reported for *Des* knockout mice. *Des-*null mice exhibit lesions on the exterior of the heart, most prominently toward the epicardium on the free wall of the right ventricle (Thornell et al., 1997). These lesions lack NADH-TR activity, similar to the *Hira* CKO. Like *Hira* CKO mice, *Des* knockouts exhibit compromised systolic function and increased fetal cardiac gene expression (Milner et al., 1999). An interesting similarity between these two genetic models is the observation that, although each of these genes was deleted in all cardiomyocytes, pathology preferentially manifests in the subepicardial region of the right ventricle. *Mdx* mice also acquire impaired cardiac function accompanied by fibrosis primarily in the free wall of the right ventricle (Meyers and Townsend, 2015). These observations suggest that cardiomyocytes in the right ventricle free wall are highly susceptible to stress-induced degeneration.

Although *Des* gene expression was unaltered in the *Hira* CKO, expression of other intermediate filaments or intermediate-filament-associated proteins were affected, including downregulation of several genes linked to heart disease or developmental defects (Fig. S5). In particular, vinculin (*Vcl*) mutations can cause some of the same issues. In humans, *Vcl* mutations cause dilated cardiomyopathy type 1W, which, like the *Hira* CKO phenotype, is associated with impaired systolic function and hypertrophy (Maeda et al., 1997; Olson et al., 2002; Vasile et al., 2006). In mice, CKO of *Vcl* in cardiomyocytes results in dilated cardiomyopathy, but focal replacement fibrosis was not reported (Zemljic-Harpf et al., 2007). However, even a modest reduction in vinculin causes cardiomyocyte necrosis and increased susceptibility to stress-induced cardiomyopathy, likely owing to disruption in the linkage between the sarcolemma and the actin cytoskeleton (Zemljic-Harpf et al., 2014, 2004). Thus, like the *Hira* CKO, *Vcl* deficiency seems to cause structural instability of the cardiomyocyte sarcolemma.

In summary, loss of HIRA widely altered gene expression, leaving cardiomyocytes susceptible to cellular stresses. These defects gave rise to hypertrophy, compromised sarcolemmal integrity and impaired cardiac function. Eventual cardiomyocyte degeneration was resolved by focal replacement fibrosis. The results of this study illustrate the importance of HIRA-mediated chromatin remodeling and replication-independent chromatin assembly of histone variant H3.3 in cardiomyocyte homeostasis. Importantly, this work reveals cardiac pathways sensitized to derailment in individuals with 22q11.2 deletion syndrome.

## MATERIALS AND METHODS

### Animals

*Hira^tm1a(EUCOMM)Wtsi^* (knockout first gene trap) mice were obtained from the European Mouse Mutant Archive (EMMA, stock #EM:05901). *Rosa26^YFP/YFP^* and *αMyHC-cre^Tg/+^* mice were obtained from the Jackson Laboratory (stock #7903 and #11038, respectively). The *Hira^flox^* allele was generated by crossing *Hira^tm1a(EUCOMM)Wtsi^* to *Rosa26^FLP/FLP^* (Flipper) (Jackson Laboratory, stock #9086). *Hira^+/−^* mice were generated by crossing *Hira^flox/+^* to the maternal deleter *Tg(Sox-2-cre)* (Jackson Laboratory, stock #8454). Experimental animals were generated by crossing *αMyHC^cre/+^; Hira^flox/−^* mice to *Hira^flox/flox^; Rosa26^YFP/YFP^* mice. Controls were *αMyHC^cre/+^; Hira^flox^^/+^; Rosa26^YFP/+^. Hira* CKOs were *αMyHC^cre/+^; Hira^flox/−^; Rosa26^YFP/+^*.

The following primers were used for genotyping: Cre-F: 5′-GCCACCAGCCAGCTATCAACTC-3′, Cre-R: 5′-TTGCCCCTGTTTCACTATCCAG-3′, Hira-F: 5′-CCTTCCTCTGCTTTGTTTGTTC-3′, Hira-R2: 5′-CCACCGCACACAGTTCACAC-3′, Hira-R3: 5′-GCCAAGTGAGCACAGAAGATGG-3′. Hira-F/R2 identifies wild-type and flox alleles (691 bp and 783 bp, respectively). Hira-F/R3 identifies the null allele (587 bp). Mice were euthanized by CO_2_ inhalation followed by cervical dislocation or by bilateral thoracotomy following P-V loop experiments. All experimental procedures involving mice were approved by the Institutional Animal Care and Use Committee of the University of Houston.

### Tissue collections

Hearts from 6-week-old mice were dissected in PBS immediately after euthanasia and flushed clean of blood by aortic injection of PBS. For RNA or protein extraction, tissues were flash frozen in liquid nitrogen immediately after dissection and stored at −80°C. For immunofluorescence, EBD fluorescence, and TUNEL and NADH-TR staining, tissues were flash frozen in liquid-nitrogen-cooled isopentane and stored at −80°C for cryosectioning.

Tissues used for hematoxylin and eosin (H&E) and Masson's trichrome staining were those collected following P-V loop assays. Briefly, we retrograde-perfused the harvested adult mouse hearts through the aorta using a Langendorff perfusion apparatus with 10% formalin for 15 min and then immersion-fixed overnight with the same solution. Following fixation, tissues were dehydrated through an ethanol gradient and embedded in paraffin for histological sections.

### Histology and immunofluorescence

H&E and Masson's trichrome staining were performed by standard methods using 7-µm paraffin sections. Immunofluorescence was performed on 7-μm cryosections using antibodies for cardiac troponin T (Developmental Studies Hybridoma Bank, clone RV-C2), γ-H2A.X (Cell Signaling, cat. #9718), nitrotyrosine (Santa Cruz Biotech, cat. #sc-32757), smooth-muscle α-actin (Sigma, cat. #C6198) or β-MyHC (Developmental Studies Hybridoma Bank, clone A4.840). Sections were blocked and antibodies diluted using 1× Casein-10%-normal serum-PBST (10× Casein stock and normal serum were from Vector Laboratories, Burlingame, CA). Primary antibodies were incubated overnight at 4°C. Secondary antibodies were incubated for 1 h at room temperature. Slides were stained with DAPI during washing and coverslips mounted with VECTASHIELD HardSet mounting medium (Vector Laboratories).

### Minimum Feret diameter measurements

Cardiomyocyte minimum Feret diameter was calculated using the analyze particles tool of ImageJ software. Data from 15- and 25-day-old mice were derived from 20× composite (large scan) WGA-488 fluorescence images. In total, 126-427 cardiomyocytes were measured per animal depending on the image size (*n*≥5 animals/group). Data from 6-week-old mice were derived from individual 40× WGA-488 fluorescence images. A total of 33-181 cardiomyocytes were measured per animal depending on image size (*n*≥6 animals/group). Data from 6-month-old mice were derived from 20× composite (large scan) H&E images. In total, 115-457 cardiomyocytes were measured per animal depending on the image size (*n*≥5 animals/group).

### NADH-TR staining

Cryosections (7 μm) were stained for NADH-TR by standard methods. The oxidative reaction was performed for 15 min at room temperature.

### BrdU and TUNEL assays

To assay cell proliferation, mice were injected with 10 µl/g body weight BrdU labeling reagent (Invitrogen, cat. #00-0103) 4 h before sacrifice. Immunofluorescence was performed as described above with anti-BrdU monoclonal antibodies (Developmental Studies Hybridoma Bank, clone G3G4). Apoptosis was assayed using the Click-iT^®^ Plus TUNEL Assay kit (Life Technologies, cat #C10618) according to the manufacturer's instructions.

### EBD uptake assay

Mice were given EBD (5 μl of 1% solution in PBS/g body weight) by intraperitoneal (IP) injection and sacrificed 18 h later for tissue collection. EBD staining was viewed as far-red fluorescence in 10-μm cryosections.

### Microscopy

Brightfield and epifluorescence images of tissue sections were obtained using a Nikon Ti-E inverted microscope equipped with a DS-Fi1 5-megapixel color camera (Nikon Instruments), a CoolSNAP HQ2 14-bit monochrome camera (Photometrics, Tucson, AZ) and NIS Elements software v4.13 (Nikon Instruments). Stereoimages (brightfield and fluorescence) were captured with a Leica MZ10F stereomicroscope and the extended depth-of-focus feature of LAS v3.7 software (Leica Microsystems, Wetzlar, Germany).

### Western blotting

Protein samples for western blot assays were extracted from snap-frozen ventricular tissues by homogenization in tissue protein extraction reagent (T-PER) buffer (Life Technologies, Carlsbad, CA) supplemented with 1 mM phenylmethanesulfonyl fluoride (PMSF), 1 mM NaVO_4_, 10 mM NaF, 1 mM sodium pyrophosphate and 1× cOmplete protease inhibitors (Roche, cat. #4693116001) (20 ml buffer/g of tissue). The suspension was centrifuged at 10,000 ***g*** for 5 min and the supernatant collected. Protein concentrations were measured by Bradford assay (Bio-Rad Protein Assay, Bio-Rad Laboratories, Hercules, CA) following standard methods. 30 μg of protein was solubilized in Laemmli buffer, heated at 90°C for 5 min and separated by 4-20% SDS-PAGE (Mini Protean II System, Bio-Rad Laboratories). Proteins were transferred onto 0.2-μm nitrocellulose membranes (Bio-Rad Laboratories). Membranes were blocked with 5% nonfat milk-TBST for 1 h at room temperature. Primary antibodies recognizing MnSOD (Santa Cruz Biotech, cat #sc-30080) or β-actin (Santa Cruz Biotech, cat #sc-1616) were diluted in 2.5% nonfat milk-TBST. Primary antibodies were incubated with the membrane overnight at 4°C, washed with TBST, and then incubated with the appropriate horseradish-peroxidase-conjugated secondary antibody for 1 h at room temperature. Membranes were washed again, and visualized by chemiluminescence using SuperSignal West Pico Chemiluminescent Substrate (Thermo Scientific, Rockford, IL).

### ROS accumulation assay

Snap-frozen ventricular tissues were homogenized in 50 mM phosphate buffer (pH 7.2) containing 1 mM ethylenediaminetetraacetic acid (EDTA), phosphatase inhibitors and protease inhibitors. Homogenates were centrifuged at 900 ***g*** for 15 min at 4°C. Supernatants were collected and centrifuged again at 16,000 ***g*** for 15 min at 4°C. The resulting supernatants were used for the assay. Protein concentrations were determined by Bradford assay. 25 μg of extract was diluted in the same buffer used for homogenization containing a final concentration of 25 μM 2,7-dichlorodihydrofluorescein diacetate (Cayman Chemical, cat. #85155). Samples were incubated for 30 min at 37°C. Fluorescence (excitation 485 nm, emission 530 nm) was measured using a SpectraMax M5 plate reader (Molecular Devices, Sunnyvale, CA).

### Real-time PCR

Total RNA for qPCR was extracted using TRIzol reagent according to the manufacturer's protocol (Life Technologies, Carlsbad, CA). Relative mRNA levels were measured using TaqMan gene expression assays with 6-carboxyfluorescein (FAM)-labeled probes. Primer probe sets were: Mm01319006_g1 for *Myh7* (Life Technologies) and custom oligos for *Myh6* (Myh6-F: 5′-GCTGACAGATCGGGAGAATCAG-3′, Myh6-R: 5′-TGCAATGCTGGCAAAGTACTG-3′, Myh6-Probe: 5′-TCCTGATCACCGGAGAATCCGGAG-3′) and *Gapdh* (Gapdh-F: 5′-ACTGGCATGGCCTTCCG-3′, Gapdh-R: 5′-CAGGCGGCACGTCAGATC-3′, Gapdh-Probe: 5′-TTCCTACCCCCAATGTGTCCGTCGT-3′) (Biosearch Technologies, Petaluma, CA). *Gapdh* mRNA expression levels were used for normalization. The PCR was run using an ABI Prism 7900HT thermocycler and SDS2.1 software (Applied Biosystems). Data were analyzed by the comparative ΔΔCT method.

### Microarrays

Total RNA for microarray analysis was extracted from non-scarred free-wall left-ventricular tissues of both control and *Hira* CKO mice (*n*=4 mice per group) using TRIzol reagent (Life Technologies) according to the manufacturer's instructions. Each sample was hybridized to triplicate arrays. Gene expression profiling and data analysis was performed using the MouseOne Array Plus v2.1 service from Phalanx Biotech (Palo Alto, CA). Raw intensity data was normalized using the median scaling normalization method. Standard selection criteria to identify differentially expressed genes were as follows: (1) log2 fold change ≥1 and *P*<0.05 or (2) log2 fold change not available and the absolute intensity difference between the two samples ≥1000. Cluster analysis was performed on a subset of differentially expressed genes. An intensity filter was used to select differentially expressed genes where the difference between the maximum and minimum intensity values exceeded 700 among all microarrays. This yielded 275 genes. An unsupervised hierarchical clustering analysis on these 275 genes revealed the correlation of expression profiles between samples and treatment conditions. PCA was also performed on this subset of genes to evaluate any differences among biological replicates and their treatment conditions. Gene ontology (GO) analyses were performed using DAVID (https://david.ncifcrf.gov/). Enriched GO terms were ranked by *P*-value. The dataset was deposited to the NCBI Gene Expression Omnibus (GEO) database under series accession number GSE71833 (http://www.ncbi.nlm.nih.gov/geo/query/acc.cgi?acc=GSE71833).

### P-V loop measurements

*In vivo* left ventricular P-V loops were measured as previously described (Pacher et al., 2008). Mice were anesthetized with isoflurane (1.5% to 2% isoflurane mixed with 100% oxygen). Briefly, a pressure-conductance catheter (PVR-1045; Millar Instruments) was inserted into the left ventricle through the right carotid artery. Steady-state P-V loops were recorded at baseline and during inferior vena cava (IVC) occlusions to cause a progressive fall in preload. Catheter calibrations were performed using the hypertonic saline and heparinized blood cuvette calibration method.

### Statistical analyses

For experiments with more than two experimental groups, quantitative data were subjected to one-way analysis of variance (ANOVA) using MedCalc v14.12.0 software (MedCalc Software bvba, Belgium). If the ANOVA was positive (*P*<0.05), a post-hoc Student–Newman–Keuls test was performed for pairwise comparison of subgroups. For studies with two experimental groups, an independent samples Student's *t*-test was performed. A *P*-value of <0.05 was considered significant for all tests.
